# Rheumatism and Gout

**Published:** 1905-12-30

**Authors:** 


### Progress in Medicine and Surgery.

### RHEUMATISM AND GOUT.

**Causes of Arthritis.**—L. W. Eley,[1] remarks that "rheumatism in the foot" is generally due to something else than true rheumatism. Pain in the foot is often produced by a weak or flat foot in persons who have to stand much. Here there may be no deformity, or there may be flattening, or abduction, or fixation in the latter position. Metatarsalgia, a neuritis affecting the nerve between two of the metatarsal bones is another cause of pain. Gonorrhœal arthritis of the ankle, and an ill-defined affection of the feet from the same cause with great sensitiveness in the soles, tubercular disease, old fractures either of the Potts' variety or those affecting the os calcis or astragalus, may all be mistaken for true rheumatism. Sometimes, too, the same mistake is made as to cases of locomotor-ataxy, or even as to infantile paralysis, gout, or the pains of late syphilis. Any of these affections may lead to humiliating errors in diagnosis, though they are usually not difficult to distinguish. Eley recommends for flat foot careful strapping with exercises and pads, and indeed strapping may be employed with advantage for several of these affections. C. C. Ransom[2] points out that one difficulty in diagnosing the cause of an arthritis is that we may first see it at a late stage when several joints are involved and the picture of the disease is no longer typical. Indeed, the situation of the affected joint or joints affords little help, as he shows in detail, but he thinks that for the diagnosis of true rheumatism salicin given in 20-grain doses every five hours, or in smaller doses for three days, is reliable; while in gout the same help can be obtained by the use of colchicin. In case of doubt, material improvement will be effected in a few days by salicin if the arthritis is rheumatic in origin, and by colchicin if it is due to gout.

**Therapeutics.**—Menzer's rheumatic serum was tested by Schaefer[3] in four chronic cases which were cured in five days. A writer in the *Medical Record* claims great success from the injection of 8 drops of a $2\frac{1}{2}$ per cent. solution of formic acid in 10 or 12 places. The injection just under the skin of a few drops of 1 per cent. of cocaine some minutes before will prevent any pain, and the formic acid may be introduced into the same punctures as soon as they are anæsthetic. He finds the treatment of great value in lumbago, sciatica, and various forms of arthritis. J. Burnet[4] gives his experiences with aspirin, which he thinks should replace the other salts of salicyclic acid, as he believes it is a more powerful analgesic and antithermic, which does not irritate the stomach, and is more pleasant to take. He thinks the rarity with which it causes tinnitus is due to the slowness of its absorption. Another advantage is that it does not depress the heart. He points out that if we give it with an alkali its special value is destroyed, for it then liberates salicylic acid in the stomach, or even in the bottle. Lemon water or cold milk forms a suitable vehicle. The dose in acute rheumatism is 10 or 15 grains every four hours, and it is valuable in lumbago, pleurodynia, rheumatic sore throat, and mild types of diabetes. The slowness of absorption, however, seems somewhat problematical, for it can be detected in the urine within an hour after being taken. Again, the gastric irritation, which he refers to as sometimes produced by salicylates, is in some instances due to insufficient dilution, and can then hardly be charged to the drug itself. The same writer also speaks highly in favour of mesotan as an application for muscular rheumatism. To avoid irritating the skin it is necessary to mix the mesotan with an equal quantity of olive oil, and merely to paint on the mixture and not rub it in, choosing a different place on each occasion. Moreover, as mesotan is decomposed by water, it should be kept dry, the perspiration should be removed from the skin first, and no impermeable material should be placed over it.

**Rheumatic Myositis.**—Norstrom[5] and othera dvo cates of massage lay much stress on the presence of nodes or indurations in muscles especially near their insertions, due to what is called muscular rheumatism. At first there is only perceived a sensation of decreased elasticity or doughiness; but as time goes on this is replaced by definite induration, and severe pain is felt on movement. The condition is often mistaken for infiltrations of the skin, for enlarged glands, pleurisy, intercostal neuralgia, and even for intra-abdominal affections, kidney disease, or neuritis. Torticollis and headaches may be due to myositis in the muscles of the neck, such as the trapezius, as well as some cases of lumbago and even pains in the abdominal walls. A favourite situation, too, is the insertion of the deltoid muscle, whence the pain radiates up and down the arm. Indeed, Norstrom has found it in all the muscles of the arm except the biceps. Sciatica is frequently complicated with myositis, which generally attacks either the gluteus medius or the peronei muscles. The quadriceps femoris, the adductors, and the muscles in the neighbourhood of the tendo Achillis are also favourite seats of the affection. Long continued, careful massage of the indurated spot appears to give a permanent cure in all these cases. The treatment of the neuritis of the arm or leg in these rheumatic patients is often a difficult matter, and there is often some joint trouble associated with it. A. W. Sikes[6] thinks that when the acute stage is over meat may be given with advantage, and that carbohydrates should be restricted. Alkaline lotions, electric light and heat baths are of value even in early stages, and at a later time the high frequency and the sinusoidal currents or the anode of the constant current may be used as well.

**The Pathology of Gout.**—Among recent theories one of the most interesting is that of the combination in health of uric acid with thyminic acid, and a good account of it is given by R. Fenner.[7] As all uric acid is derived from nucleic acid, which affords at the same time a supply of thyminic acid, rendering it freely soluble, it gives as a rule no trouble to the organism. Indeed the combination is so firm that uric acid cannot be detected in the blood in health, though it is constantly excreted, and is not manufactured in the kidneys. In gout, however, uric acid can be found in the serum, and the question is why in gout there is not a supply of thyminic acid as in health to combine with it? For if thyminic acid is given to gouty patients the uric acid readily unites with it. It is possible that in gout uric acid is evolved from some new source which does not afford the other factor, that it is a synthetic product, and not as in health due to the oxidation of nucleins or their derivatives the purin bases. Certainly the administration of thyminic acid to the gouty leads to an enormous increase in the excretion of uric acid, while in health it has no effect. On the other hand, the objections have been made to this theory, that normal blood does contain appreciable amounts of urates, and that the formation of a synthetic product, such as this uric acid in gout, is contrary to the course of all ordinary metabolism; and lastly, that there is no direct proof of the circulation of the combined uric and thyminic acids. Fenner records great success from the use of tablets of thyminic acid both in acute and chronic gout. An attempt to render uric acid freely soluble by the use of formaldehyde may be compared with this. Merkel[8] has used citarin, a compound of formaldehyde and sodium citrate, and reviews the reports of other writers. He finds that if it is administered quickly and in large doses it cuts short acute attacks rapidly. He gave usually four doses of 30 grains each on the first day, and three on each of the next three days, after food. The drug, too, should be given in cold water without the addition of other substances, and appears to be harmless. Chronic cases show less benefit. Another theory of gout, expounded by Francis Hare, late of Brisbane, ascribes it to an excess of hydrocarbons absorbed. In health they are either stored up as fat or burnt off as carbon dioxide, but pathologically they may remain in the blood in excess until they lead to attacks of asthma, acute gout, epilepsy or other disturbances. Gout does not occur on a purely proteid diet, for that limits the supply of hydrocarbons, nor on a non-nitrogenous one, for that prevents absorption. Thus we find two forms of diet recommended in gout; one in which proteids are cut down to the minimum, and the other, which consists of meat and green vegetables, in which the supply of carbohydrates is reduced. Again, an excess of hydrocarbons leads to an accumulation of uric acid in the blood. When this becomes very great the kidneys can no longer deal with it, until an acute arthritis has burnt up the excess of hydrocarbons, and then once more the uric acid is freely excreted by the kidneys. Thus the acute attack is nature's method of removing the excess of carbonaceous matter. As a theory the argument is interesting, but proof of this excess of hydrocarbons and of its relation to excess of uric acid is wanting. Ringrose Gore, on the other hand, is a thorough-going opponent of the uric acid theory, denying that it ever causes any of the symptoms or has any toxic properties. The deposit of biurate in a joint is not, he says, the cause, but a harmless result, of the inflammation, which subsides, though the unirritating deposit remains there. In leukæmia far greater amounts of uric acid exist in the blood than in gout, and yet there are no symptoms. Now the common cause of joint inflammation is some bacterial toxin, and, as in gout, there is usually present, too, some irritation of the skin and mucous membranes which excrete such poisons from the system. He believes that some poison is formed in the intestinal canal which causes the symptoms, and that this poison is not produced by excessive absorption of some element of food, but by intestinal catarrh increasing the virulence of some bacterial toxin normally produced there. Even in health 9 grams of bacteria are found in the fæces daily, and this amount is increased either by an excess of proteid food and alcohol or of farinaceous food and tea. Thus the diets which are said to increase uric acid are those which favour bacterial growth, while the administration of uric acid has no toxic effect whatever; especially it does not produce the symptoms of gout. Again, indican appears in gout, and disappears under treatment, and it is known that this is due to bacterial growth in the upper part of the intestine which should normally be sterile. Colchicum, too, which relieves gout, acts primarily on the intestinal secretions, and lead, which provokes it, diminishes them. Indeed, success in treatment depends on the production of a healthy gastric intestinal tract. Turning to treatment, in an acute attack he gives 20 minims of colchicum with potash, followed by 15 minims every two or three hours till the pain is relieved, together with calomel and saline aperients. Plenty of water may be taken, but either no food at all for 48 hours or only soda and milk or whey. A milk diet should, he says, be continued for weeks, and if there is heart failure ammonia and nux vomica are preferable to alcohol. When convalescent the diet to which a patient is accustomed should not be suddenly changed, since the secretions are regulated to the habitual food; but meat, alcohol, tea, sugar, and indigestible vegetables should be carefully restricted.

[1] Med. Rec., Aug. 5. [2] Med. Rec., June 10. [3] Amer. Jour. Med. Sciences, March. [4] Lancet, May 6. [5] Med. Rec., Mar. 11. [6] Clin. Jour., Aug. 2. [7] Lancet, July 1. [8] Clin. Excerpts, Nov.-Dec. [9] Med. Rec., June 17. [10] Clin. Jour., July 26. [11] Lancet, June 17.

(*To be concluded.*)

